# Design and Analysis of Rhesus Cytomegalovirus IL-10 Mutants as a Model for Novel Vaccines against Human Cytomegalovirus

**DOI:** 10.1371/journal.pone.0028127

**Published:** 2011-11-21

**Authors:** Naomi J. Logsdon, Meghan K. Eberhardt, Christopher E. Allen, Peter A. Barry, Mark R. Walter

**Affiliations:** 1 Department of Microbiology, University of Alabama at Birmingham, Birmingham, Alabama, United States of America; 2 Center for Comparative Medicine, University of California Davis, Davis, California, United States of America; Bioinformatics Institute, Singapore

## Abstract

**Background:**

Human cytomegalovirus (HCMV) expresses a viral ortholog (CMVIL-10) of human cellular interleukin-10 (cIL-10). Despite only ∼26% amino acid sequence identity, CMVIL-10 exhibits comparable immunosuppressive activity with cIL-10, attenuates HCMV antiviral immune responses, and contributes to lifelong persistence within infected hosts. The low sequence identity between CMVIL-10 and cIL-10 suggests vaccination with CMVIL-10 may generate antibodies that specifically neutralize CMVIL-10 biological activity, but not the cellular cytokine, cIL-10. However, immunization with functional CMVIL-10 might be detrimental to the host because of its immunosuppressive properties.

**Methods and Findings:**

Structural biology was used to engineer biologically inactive mutants of CMVIL-10 that would, upon vaccination, elicit a potent immune response to the wild-type viral cytokine. To test the designed proteins, the mutations were incorporated into the rhesus cytomegalovirus (RhCMV) ortholog of CMVIL-10 (RhCMVIL-10) and used to vaccinate RhCMV-infected rhesus macaques. Immunization with the inactive RhCMVIL-10 mutants stimulated antibodies against wild-type RhCMVIL-10 that neutralized its biological activity, but did not cross-react with rhesus cellular IL-10.

**Conclusion:**

This study demonstrates an immunization strategy to neutralize RhCMVIL-10 biological activity using non-functional RhCMVIL-10 antigens. The results provide the methodology for targeting CMVIL-10 in vaccine, and therapeutic strategies, to nullify HCMV's ability to (1) skew innate and adaptive immunity, (2) disseminate from the site of primary mucosal infection, and (3) establish a lifelong persistent infection.

## Introduction

Human cytomegalovirus (HCMV) is a ubiquitous human β-herpesvirus (50->95% adult seroprevalence worldwide) that can infect a susceptible individual at any time during pre- or post-natal life [1]. HCMV infection is generally subclinical in those with functional immune systems. However, HCMV establishes and maintains a lifelong persistence despite a robust host immune response. In fact, ∼10% of memory CD4^+^ and CD8^+^ T-cells in long-term infected hosts are HCMV-specific [2], and generate antibodies against multiple HCMV glycoproteins that neutralize the virus [3], [4]. Persistence is characterized by the presence of cells harboring essentially quiescent HCMV genomes that can asymptomatically reactivate to produce infectious virions that can be shed in bodily fluids, such as breast milk, saliva, and urine.

Serious HCMV-induced clinical outcomes can occur in those with immature or compromised immune systems, including congenitally infected newborns, immunosuppressed transplant recipients, and immunodeficient AIDS patients [5]. Transplacental transmission from mother to fetus can occur during primary HCMV infection of the mother, reactivation of persistent virus within the mother, or maternal re-infection. In the case of maternal re-infection, the demonstration that 10% of seropositive women who give birth to a congenitally infected infant acquired new antigenic reactivity to HCMV antigens between pregnancies is indisputable evidence that prior immunity is incompletely protective against reinfection with antigenic HCMV variants [6]. These results further suggest that reinfection with HCMV leads to attenuation of antiviral effector/memory functions, enabling progeny virions to ultimately disseminate beyond the mucosal site of reinfection to the maternal/fetal interface. In both solid organ (SOT) and bone marrow transplantation (BMT), resident HCMV genomes can reactivate under conditions of iatrogenic immunosuppression. For HIV-infected individuals, resident HCMV genomes can reactivate during onset of immunodeficiency and cause end-organ disease, such as retinitis [7].

Since HCMV was recognized as an infectious threat to the fetus, there have been repeated calls for a vaccine that prevented congenital infection in women without preconceptional immunity to HCMV [8], [9], [10], [11]. The advent of solid organ and bone marrow transplantation as medical options has heightened the need for an HCMV vaccine to protect immunosuppressed recipients from fulminant HCMV infections. Progress on a vaccine has been made using glycoprotein B (gB) in clinical trials designed to protect seronegative women with children from primary infection, and seronegative transplant recipients from HCMV infection and/or disease post allograft [12], [13]. Both trials achieved measurable (∼50%) successes in reducing the rate of acquisition of HCMV, the extent of HCMV replication and length of anti-HCMV drug therapies, respectively. The absence of complete protection in both trials argues that further vaccine optimization is required to eliminate the risk of pathogenic outcomes associated with HCMV infection, re-infection, and/or reactivation. One reason for sub-optimal performance of the current gB-vaccines may be the absence of other, viral proteins that could increase vaccine-mediated protective efficacy. One such class of viral proteins that has not been investigated consists of the HCMV-encoded immuno-modulatory proteins that are thought to be critical viral elements responsible for attenuation of host immunity *in vivo* [14].

The HCMV IL-10 ortholog, CMVIL-10, is one such viral immune modulating protein that offers several potential advantages for vaccination. Despite only 26% amino acid sequence identity between CMVIL-10 and cellular IL-10 (cIL-10), CMVIL-10 retains the immunosuppressive properties of cIL-10 on multiple lymphoid cell types, especially dendritic cells (DC), which link innate and adaptive immunity [15], [16], [17], [18], [19]. In addition, cIL-10 and CMVIL-10 both engage the IL-10R1 and IL-10R2 cell surface receptor chains to induce their biological activities [19], [20], [21], [22]. Binding studies demonstrate cIL-10 and CMVIL-10 form similar high affinity (∼1 nM) interactions with the IL-10R1 chain and low affinity (∼µM) contacts with the IL-10R2 chain [22], [23]. As a result, the IL-10/IL-10R1 interaction occurs first, followed by the assembly of the IL-10/IL-10R1/IL-10R2 ternary complex, which activates intracellular kinases (Jak1 and Tyk2) and transcription factors (STAT3) leading to IL-10 cellular responses [19]. The importance of viral IL-10 *in vivo* is highlighted by a recent study showing that primary infection of rhesus (Rh) macaques with a variant of RhCMV lacking the RhCMVIL-10 gene led to (1) increased innate responses at the site of inoculation, and (2) increased long-term B and T cell responses to RhCMV antigens, compared to infection with the parental variant expressing RhCMVIL-10 [15]. These studies suggest a mechanism by which early interactions between viral IL-10 and DC at the site of infection skew the adaptive responses to a state favoring viral persistence. The precise role of CMVIL-10, relative to cIL-10, remains to be determined. However, studies show CMVIL-10 induces cIL-10 in DC and trophoblasts and prevents effective T-cell priming by inhibiting dendritic cell (DC) maturation and priming [16], [24]. Furthermore, the CMVIL-10 open reading frame (ORF) is conserved in numerous culture-adapted strains and clinical isolates of HCMV [25]. Together, these data suggest CMVIL-10 is an attractive target for vaccine development.

To test this hypothesis in an animal model, we sought to use RhCMVIL-10 as an antigen for vaccination studies in rhesus macaques (*Macacca mulatta*). The use of the RhCMV model was essential since, in contrast to murine CMV, RhCMV encodes a viral IL-10 (RhCMVIL-10) and displays similar infection routes, seroconversion rates, and sheds virus in urine and saliva as observed for HCMV [26]. However, since RhCMVIL-10 exhibits potent immunosuppressive activities that disrupt immune function, wild-type (WT) RhCMVIL-10 was not appropriate for our studies. To disrupt RhCMVIL-10 biological activity, we designed two RhCMVIL-10 mutants, guided by crystal structures of the cIL-10/IL-10R1 and CMVIL-10/IL-10R1 complexes that could not bind IL-10R1 [21], [27]. The designed mutants fail to induce RhCMVIL-10 biological activities. Vaccination of RhCMVIL-10-infected rhesus macaques with the inactive RhCMVIL-10 mutants generated anti-RhCMVIL-10 Abs that block the biological activity of wild-type RhCMVIL-10 and do not cross react with cellular RhIL-10.

## Results

### Expression, purification, and characterization of RhCMVIL-10

**Figure 1:**
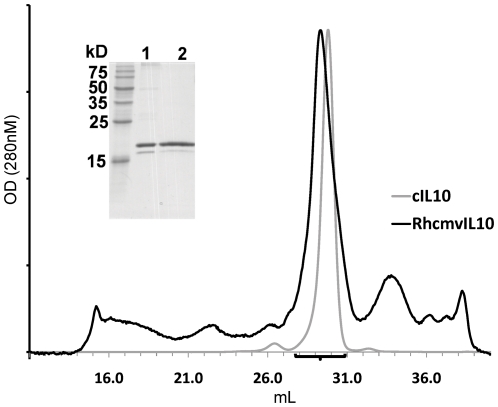
Purification and quaternary structure of RhCMVIL-10. GF Chromatographs of cIL-10 (grey) and RhCMVIL-10 (black) plotted with X-axis in mL and Y-axis in optical density (OD) at 280 nm. (Inset) SDS-PAGE gel of affinity purified RhCMVIL-10 (lane 1) and pooled fractions (black bar) of the major RhCMVIL-10 GF peak (lane 2).

**Figure 2:**
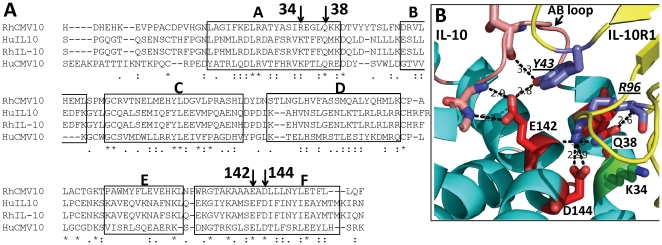
Sequence and structure model of RhCMVIL-10 binding residues. (A) Sequence alignment of cellular and viral human and rhesus IL-10s. The predicted site for N-linked glycosylation in RhCMVIL-10 is underlined. GenBank Accession numbers: RhCMVIL-10, AAF59907; cIL-10, AAA63207; RhIL-10, AAA99975; CMVIL-10, AAF63437. CIL-10 helices are denoted on the alignment (A–F [28]), which also highlights residues chosen for mutagenesis. (B) Structure model of the RhCMVIL-10/RhIL-101R1 interface based on the crystal structure of the HuIL10/HuIL-10R1 complex (pdbid 1Y6K, [23] ). Residues chosen for mutagenesis that disrupt IL-10R1 binding are shown in red, while the Arg-34Glu mutant (corresponding to Lys-34 in CMVIL-10) that exhibits essentially WT activity is colored green (see Fig. 4A).

RhCMVIL-10 was expressed in Drosophila Schneider S2 cells and purified by affinity chromatography using agarose beads coupled with the human IL-10R1 chain as previously described [21]. Affinity purified RhCMVIL-10 ran as two bands on SDS-PAGE gels. The major band exhibited a molecular weight (MW) of ∼19 kilodaltons (kDa), while a much less intense second band of ∼18 kDa was also observed (Fig 1). The presence of two bands suggested RhCMVIL-10, expressed in insect cells, is predominantly glycosylated on its single N-linked glycosylation site, Asn-87 (Fig 2A). Affinity purified RhCMVIL-10 fractionated from a size exclusion column at essentially the same position as cIL-10, which was previously shown to form a noncovalent homodimer [28], [29], [30]. The combined results of the SDS-PAGE and GF data are most consistent with the interpretation that RhCMVIL-10 is a glycosylated non-covalent homodimer.

### Design of RhCMVIL-10 point mutants defective in IL-10R1 binding

Crystal structures of the cIL-10/IL-10R1 and CMVIL-10/IL-10R1 complexes, combined with sequence analysis, were used to assist in the design of RhCMVIL-10 point mutants that could not bind to the IL-10R1 chain (Fig 2). Mature cIL-10 and RhIL-10 sequences share 95% sequence identity, while the viral IL-10s (RhCMVIL-10 and CMVIL-10) exhibit between 23%-28% identity with each other and with the cellular IL-10s. Despite the divergent amino acid sequences, five surface exposed residues (helix A residues Arg-27, Gln-38; and helix F residues Glu-142, Asp-144, and Glu-151) are conserved in all four sequences and form extensive contacts with IL-10R1 in the cIL-10/IL-10R1 and CMVIL-10/IL-10R1 binding interfaces (Fig. 2) [21], [27]. The conserved interactions made by these five residues suggested the crystal structures of the cIL-10/IL-10R1 and CMVIL-10/IL-10R1 provide suitable structural models for selecting mutations that disrupt RhCMVIL-10/RhIL-10R1 interactions. The structural data are critical, since the goal is to design biologically inactive RhCMVIL-10 mutants that preserve the wild-type RhCMVIL-10 three-dimensional structure.

Using the structural information described above (Fig 2B), mutations of three RhCMVIL-10 residues (Gln-38, Glu-142, and Asp-144) were predicted to maximally disrupt RhCMVIL-10/RhIL-10R1 interactions and prevent RhCMVIL-10 biological activity. These residues were chosen based on the following criteria. 1) The residues were conserved among the human and viral IL-10 amino acid sequences, including CMVIL-10 from different HCMV strains; 2) the amino acids made extensive contacts with IL-10R1 in the cIL-10/IL-10R1 and CMVIL-10/IL-10R1 complexes; and 3) the residues were located in the center of the IL-10/IL-10R1 interfaces. This final criterion was required because RhCMVIL-10 mutations predicted to disrupt IL-10R1 binding, but located on edge of the IL-10/IL-10R1 interface, might assume altered side-chain conformations that could still enable efficient IL-10R1 binding. To test this latter hypothesis, an Arg-34Glu RhCMVIL-10 mutant was made. The corresponding amino acid in cIL-10 and CMVIL-10 is Lys-34, which forms salt-bridge interactions with IL-10R1 residues Asp-100 and Glu-101 on the edge of the IL-10/IL-10R1 interface (Fig 2B). As described for Arg-34Glu, amino acid changes at the identified positions were chosen to sterically prevent RhCMVIL-10/IL-10R1 complex formation and/or disrupt extensive hydrogen bonding networks found in the interface.

### Characterization of RhCMVIL-10 mutant binding to IL-10R1

**Figure 3:**
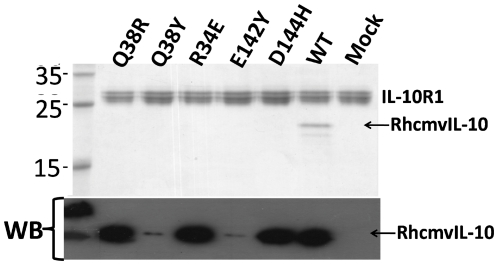
Expression and IL-10R1 binding of RhCMVIL-10 point mutants. Expression of RhCMVIL-10 point mutants in Drosophila cell media was characterized by western-blotting (WB). Cell supernatants containing the point mutants were incubated with HuIL-10R1 coupled beads. After washing, the beads were loaded onto a 12% SDS-PAGE gel and subsequently stained with coomassie blue.

Based on the analysis above, five RhCMVIL-10 point mutants (Gln-38Arg, Gln-38Tyr, Arg-34Glu, Glu-142Tyr, Asp-144His) were expressed in insect cells, and the supernatants tested for binding to the IL-10R1 chain (Fig 3). To assist in purification, and provide a common epitope for detecting the RhCMVIL-10 mutants, a 6-residue histidine tag (H6) and a factor Xa protease site (Fxa) was added to the N-terminus of these RhCMVIL-10 mutants (H6FXa-RhCMVIL-10). Western blot analysis revealed H6FXa-RhCMVIL-10 mutants Gln-38Tyr, or Glu-142Tyr, were not efficiently expressed (Fig. 3 bottom). The other 3 RhCMVIL-10 mutants (Arg-34Glu, Gln-38Arg, and Asp-144His) were expressed at comparable levels (Fig. 3 bottom), but were unable to bind to IL-10R1 coupled beads (Fig. 3 top). In contrast, WT RhCMVIL-10 was able to bind to IL-10R1 beads.

### Analysis of RhCMVIL-10 mutants ability to proliferate human TF1/IL-10R1 cells

**Figure 4:**
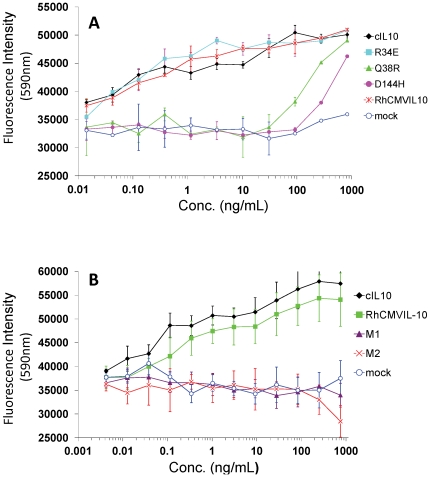
Biological activity of RhCMVIL-10 mutants M1 and M2 on TF1/IL-10R1 cells. **(A)** Cell supernatants containing RhCMVIL-10 single point mutants (described in the Figure legend) (Lys-34Glu, Gln-38Arg, and Asp-144His) were evaluated for their ability to stimulate proliferation of TF-1/IL-10R1 cells in relation to cIL-10 and WT RhCMVIL-10. **(B)** TF-1/IL-10R1 cell proliferation assay for RhCMVIL-10 M1 (Q38R, D144H) and M2 (E142Q, D144H) cell supernatants. Error bars represent estimated standard error from duplicate measurements.

To characterize the RhCMVIL-10 point mutants (Gln-38Arg, Arg-34Glu, and Asp-144His) in a more sensitive assay, we tested the ability of each mutant to stimulate proliferation of TF-1/IL-10R1 cells. TF-1/IL-10R1 cells are a human erythroleukemic cell line stably transfected with the human IL-10R1 chain, which causes them to proliferate upon addition of IL-10 to the media [31]. The concentrations of RhCMVIL-10, and RhCMVIL-10 point mutants, in Drosophila cell supernatants were estimated by SDS-PAGE. Serial dilutions of each RhCMVIL-10 mutant and cIL-10 were added to TF-1/IL-10R1 cells and proliferation was measured after 2 days. As shown in Figure 4, RhCMVIL-10 and cIL-10 exhibited essentially equivalent biological activity in the assay. RhCMVIL-10 Arg-34Glu also exhibited essentially wild-type biological activity, presumably because of its location on the edge of the IL-10/IL-10R1 interface (Fig 2). In contrast, RhCMVIL-10 Gln-38Arg and RhCMVIL-10 Asp-144His exhibited effective concentrations (EC50s) that were ∼100- and ∼300-fold lower than RhCMVIL-10 and cIL-10 (Fig. 4), but still retained considerable activity at the highest concentration tested (1,000 ng/ml).

Since the single mutants retained some activity, we tested two RhCMVIL-10 double mutations in the TF-1/IL-10R1 cell assay (Fig 4B). RhCMVIL-10 mutant 1 (M1) consisted of Gln-38Arg and Asp-144His point mutations, while RhCMVIL-10 mutant 2 (M2) contained Glu-142Gln and Asp-144His mutations. Because of the poor solubility of the H6Fxa-RhCMVIL-10 proteins during initial purification studies, a C-terminal Fxa and H6 tag (RhCMVIL-10-FXaH6) was added to RhCMVIL-10 M1 and M2. Serial dilutions of M1 and M2, based on protein concentrations estimated from SDS-PAGE gels, were added to TF-1/IL-10R1 cells, which revealed RhCMVIL-10M1 and M2 did not exhibit biological activity at concentrations as high as 750 ng/mL (Fig. 4B).

### Analysis of RhCMVIL-10 mutants ability to suppress IL-12 from Rhesus PBMC

**Figure 5:**
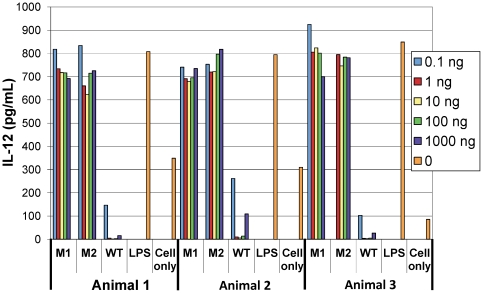
Ability of RhCMVIL-10 M1 and M2 to suppress IL-12 levels in LPS-activated rhesus PBMC. IL-12 levels produced by LPS-activated rhesus PBMCs were measured by ELISA in the presence, or absence, of purified RhCMVIL-10 M1, RhCMVIL-10 M2, or RhCMVIL-10 WT over a concentration range of 0.1–1,000 ng/ml. Also shown are results for cells incubated with media alone and with LPS alone. Assays were performed using PBMC from three rhesus macaques (Animal 1–3).

Based on the results of the TF-1/IL-10R1 assays, RhCMVIL-10 double mutants, M1 and M2, were purified by nickel affinity chromatography. Purified M1 and M2 proteins were assayed for their ability to inhibit the production of IL-12 in rhesus PMBCs stimulated with lipopolysaccaride (LPS) (Fig. 5). Addition of LPS to PBMC from three different animals stimulated high levels of IL-12 expression, relative to PBMCs incubated in media alone. In marked contrast, co-incubation of LPS-activated PBMC with WT RhCMVIL-10 strongly inhibited IL-12 expression to levels below those observed in un-stimulated cells. In contrast to WT RhCMVIL-10, RhCMVIL-10 M1 and M2 could not inhibit IL-12 production in LPS treated PBMC cultures at any concentration tested (0.1 ng/mL-1000 ng/mL). Thus, RhCMVIL-10 M1 and M2 were essentially devoid of functional activity (∼88-100% inactive, Fig. 5), as previously shown in the TF-1/IL-10R1 cell proliferation assay (Fig. 4).

### Characterization of RhCMVIL-10/IL-10R1 interactions by surface plasmon resonance (SPR)

**Figure 6:**
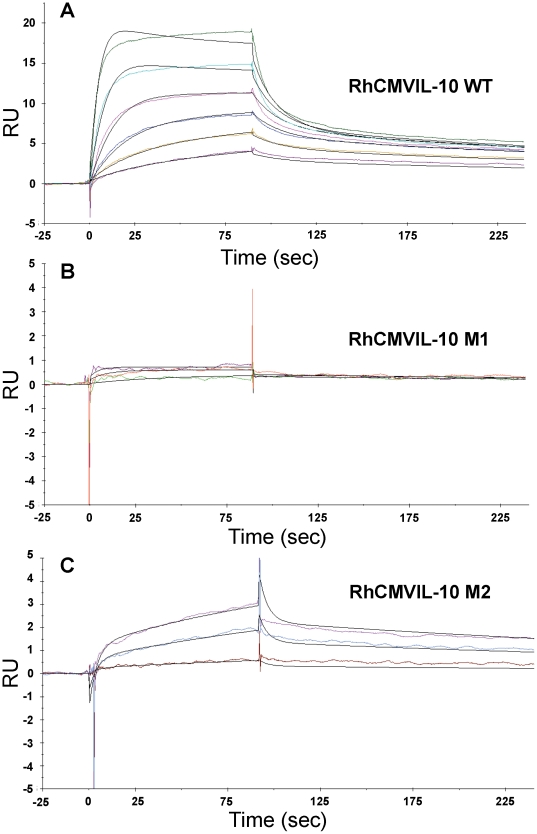
SPR analysis of RhCMVIL-10 wild-type, M1, and M2 binding to IL-10R1. Panels A, B, and C, show experimental sensorgrams (colored) and bivalent model fits (black lines) for RhCMVIL-10WT, M1, and M2, respectively. Note the Y axis in panels B and C only extends to +/- 5RU compared to −5 to 25RU in panel A. The maximum RU observed for RhCMVIL-10 M1 and M2 is below that of RhCMVIL-10WT observed at 3.125 nM; the lowest concentration tested.

Surface plasmon resonance (SPR) experiments were performed to further validate that RhcmvIL-10 M1 and M2 exhibit reduced binding to IL-10R1. The RhCMVIL-10 dimer (0–100 nM), or RhCMVIL-10 mutants (100 nM, 500 nM, and 1000 nM), were injected over a Biacore chip surface of human IL-10R1, formed by attaching an IL-10R1-FC fusion protein to a CM5 chip using an anti-FC antibody (Fig 6). Human and rhesus IL-10R1 share 94% amino sequence identity, suggesting human IL-10R1 can provide a reasonable estimate of RhCMVIL-10/RhIL-10R1 interactions. WT RhCMVIL-10/IL-10R1 sensorgrams were globally fit to a bivalent analyte kinetic model (Fig 6). The resulting binding constants reveal an initial interaction between RhCMVIL-10 and human IL-10R1 of 65.1 nM (KD1), with a second apparent binding constant, KD2, of 1.3 nM (Table 1). Interpretation of the binding constants in this kinetic model is difficult. However, they provide a baseline for comparing RhCMVIL-10 M1 and M2 binding to IL-10R1.

**Table 1:** SPR Binding Constants for RhCMVIL-10 / human IL-10R1 Interactions.

	ka1 (1/Msec)	kd1 (1/sec)	ka2 (1/RUs)	kd2 (1/sec)	Rmax (RU)	Rm-obs* (RU)	KD1 (nM)	KD2 (nM)
RhCMVIL-10	1.18E+06	0.07671	0.001055	0.00155	24.3	20.9	65.1	1.3
RhCMVIL-10M1#	4,617	0.1985	2.32E-04	0.00187	24.3	0.8	43,000	410
RhCMVIL-10M2##	1.45E+04	0.2117	5.48E-04	0.00136	24.3	3.0	14,700	94.4

Rate constants were obtained from fitting the Sensorgrams in Figure 6 to a bivalent analyte model.

**M** = molar**, RU** = Response unit, **KD1** = kd1/ka1, **KD2** = kd2/ka1, **Rmax** is the calculated Rmax determined during fitting, **Rm-obs** is the maximum RU value observed in the sensorgrams at the highest concentration collected.

# Binding parameters obtained by fixing Rmax and mass transport (tc = 1.546+E07) values to those obtained from global fitting of the RhCMVIL-10 data.

## Binding parameters obtained by fixing Rmax to the value obtained from global fitting of the RhCMVIL-10 data.

*The theoretical Rmax for RhCMVIL-10 dimer binding to IL-10R1 is 29.5RU.

RhCMVIL-10 M1 and M2 were injected at three concentrations (100 nM, 500 nM, and 1000 nM) over the IL-10R1-FC surface (Fig 6). For RhCMVIL-10 M1, the maximal response unit (RU) obtained at 1000 nM was 0.8 RU, which is 3.8% of the maximal RU obtained for RhCMVIL−10 at 100 nM (20.9 RU). KD1 and KD2 values for RhCMVIL-10M1 were estimated to be 43 µM and 410 nM, respectively (Table 1). RhCMVIL-10 M2 bound slightly better to IL-10R1 than RhCMVIL-10 M1. A maximal response of 3 RU was obtained from the 1000 nM injection of RhCMVIL-10 M2. KD1 and KD2 values for RhCMVIL-10 M2 were 14.7 µM and 94.4 nM, respectively (Table 1). Comparing the KD1 parameters suggests RhCMVIL-10 M1 and M2 exhibit at least ∼226–660 fold lower affinity for IL-10R1. However, this estimate is likely low since the impact of disrupting the second interaction in the dimer has not been considered. Thus, consistent with the bio-activity data (Figs 4 and 5), the SPR studies demonstrate the ability of RhCMVIL-10 M1 and M2 to bind IL-10R1 has been extensively disrupted, relative to wild-type RhCMVIL-10.

### Immunization of rhesus macaques with RhCMVIL-10M1 and RhCMVIL-10M2

**Figure 7:**
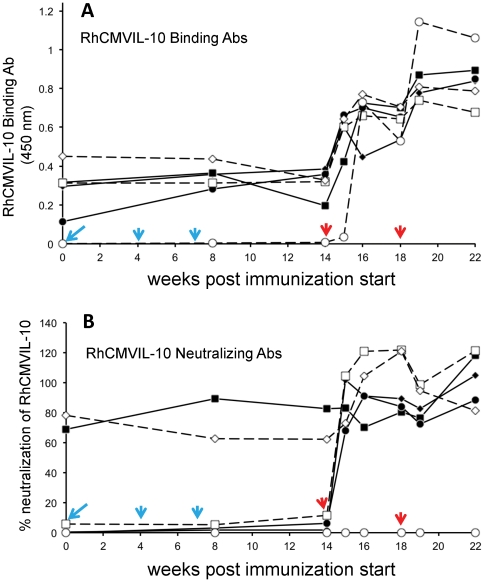
Stimulation of RhCMVIL-10 binding and neutralizing antibodies (Ab) following immunization of RhCMV-infected rhesus macaques. (A) RhCMVIL-10-binding antibodies were analyzed throughout the immunization schedule (Table 2) by ELISA measured at absorbance at 450 nm (see Methods for details). (B) Neutralization of RhCMVIL-10 WT biological activity by plasma collected throughout the immunization schedule (Table 2). Percent (%) neutralization of RhCMVIL-10 denotes the ratio ([ IL-12 ] ^Plasma+RhCMVIL-10^ / [ IL-12 ]^Plasma^ )*100, such that 100% corresponds to complete inhibition of RhCMVIL-10 and 0% is no inhibition. Values greater than 100 reflect errors/variations in the measured levels of IL-12 in the two samples. The times of DNA (cyan) and protein (red) vaccination are shown on the Figure with arrows. Three animals (A1–A3) were immunized with M1 (solid lines), and three animals (A4–A6) were immunized with M2 (dashed lines). The times of blood draws are noted by the solid symbols for M1 immunized animals and open symbols for M2 immunized animals. Where immunization and blood draws were performed on the same day, blood was taken prior to immunization. The shape of the symbols denotes different animals, with M1 immunized animals A1–A3 solid diamond, square, and circle, respectively. M2 immunized animals A4–A6 are denoted by open square, diamond, and circle, respectively. The same designations are used for panels A and B.

Based on the results of the SPR studies and the cell-based assays, RhCMVIL-10 M1 and M2 were used to immunize six RhCMV-infected rhesus macaques (Table 2) to determine whether functionally inactivated RhCMVIL-10s could generate antibodies that neutralize WT RhCMVIL-10 biological activity (RhCMVIL-10-NAbs). Immunization of RhCMV- infected animals was performed since disruption of the IL-10 signaling pathway has been proposed as a therapeutic intervention to alter the course of persistent pathogen infections (see Discussion). Thus, using infected animals, allowed us to survey their pre-vaccination RhCMVIL-10-NAb status and determine if vaccination with M1 or M2 could increase RhCMVIL-10-NAb levels. The production of RhCMVIL-10-NAbs during the immunization procedure was monitored by testing the ability of plasma, collected throughout the immunization schedule (Table 2), to antagonize RhCMVIL-10-mediated inhibition of IL-12 in LPS stimulated PBMC (Fig 7). Using this assay, the presence of RhCMVIL-10-NAbs was evaluated at the time of the initial DNA vaccination (Vx) (week 0), after the third DNA booster immunization (week 8), and six times following the two protein boosts at weeks 14 and 18 (Fig 7).

**Table 2:** RhCMVIL-10 M1 and M2 Immunization Schedule.

Week	Immunogen	Amount	route		Amount	Route	# animals	Mutant
0	DNA	150 µg	IM	&	50 µg	ID	3	M1
“	“	“	“	&	“	“	3	M2
4	DNA	150 µg	IM	&	50 µg	ID	3	M1
“	“	“	“	&	“	“	3	M2
7	DNA	150 µg	IM	&	50 µg	ID	3	M1
“	“	“	“	&	“	“	3	M2
14	Protein	50 µg	IM				3	M1
“	“	“	“				3	M2
18	Protein	50 µg	IM				3	M1
“	“	“	“				3	M2

**IM** = Intramuscular, **ID** = Intradermal.

Immunized animals did not display any detectable changes in activity, eating, or grooming, suggesting that the immunization was well tolerated. Three of the six immunized animals exhibited demonstrable RhCMVIL-10-NAb titers at the time of the first DNA immunization, ranging from 5 – 80% neutralization of RhCMVIL-10 biological activity (week 0) (Fig. 7). All six animals exhibited RhCMVIL-10 binding antibodies (BAb) at the time of the first DNA vaccination (Fig 7A). DNA immunization at weeks 0, 4, and 7 did not stimulate increases in RhCMVIL-10-NAb responses. However, RhCMVIL-10-NAb titers were prominently increased in five of the six vaccinees after the first and/or second protein vaccinations at weeks 14 and 18 (Fig 7). One vaccinee with undetectable RhCMVIL-10-NAb responses prior to immunization did not develop detectable RhCMVIL-10-NAb responses after either protein boost (Fig 7B, open circles). However, increases in BAbs, following the two protein boosts, were observed for this animal (Fig 7A, open circles). The animal that failed to respond was vaccinated with the M2 protein. While M2 might be considered a poorer immunogen than M1, based on our pre-vaccination survey (week 0), some naturally infected animals fail to develop RhCMVIL-10-NAbs, despite the presence of RhCMVIL-10-binding antibodies (Fig 7).

### Plasma from animals immunized with M1 and M2 does not cross react with cellular RhIL-10

**Figure 8:**
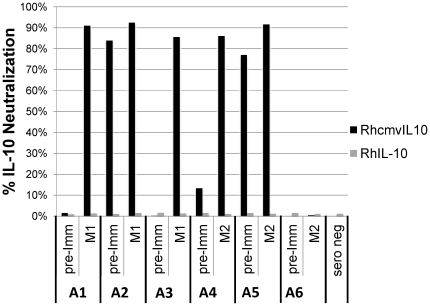
RhCMVIL-10-NAbs generated against RhCMVIL-10 M1 and M2 do not cross react with cellular RhIL-10. Plasma from animals (A1–A6) pre- (pre-Imm, week 0, Figure 7), and post-immunization (week 19 plasma sample, 1 week after the second protein boost, Figure 7), was tested for its ability to neutralize cellular RhIL-10 (grey bars) or RhCMVIL-10 (black bars) bioactivity, where bioactivity was measured as suppression of LPS-induced IL-12 production in rhesus PBMC. The data show the biological activity of RhIL-10 is not neutralized by plasma from animals immunized with RhCMVIL-10 M1 or M2. However, RhCMVIL-10 biological activity, except for A6, is almost completely neutralized by the same plasma sample. Percent (%) neutralization is calculated as described in Figure 7 and in the “Materials and methods” section.

RhCMVIL-10 shares only 23% amino acid sequence identity with RhIL-10, and there are no more that 3 contiguous amino acids that are identical when the two proteins are aligned. This suggested that RhCMVIL-10-NAbs generated by immunization with RhCMVIL-10 M1 or M2 would not cross react with cellular RhIL-10. To confirm this hypothesis, plasma samples from each animal in Figure 7, were tested for the ability to neutralize the biological activity of cellular RhIL-10 activity. This was performed by determining whether plasma samples from immunized animals that neutralized RhCMVIL-10, similarly neutralized the ability of RhIL-10 to suppress IL-12 expression in LPS-activated rhesus PBMC. As shown in Figure 8, week 19 plasma samples (Fig 7) that completely neutralized RhCMVIL-10-mediated suppression of IL-12 had no effect on RhIL-10-induced suppression of IL-12.

## Discussion

In this study, structural biology was used to rationally design non-functional RhCMVIL-10 immunogens for vaccination in a nonhuman primate model that closely mimics HCMV infection in humans. Using this approach, we have established that immunizing animals with signaling-incompetent RhCMVIL-10 mutants (M1 and M2) is able to generate, or boost, RhCMVIL-10-NAb titers in seropositive rhesus macaques. To our knowledge, this is the first report of a rational vaccine/therapeutic strategy to disrupt a viral cytokine, CMVIL-10.

Blockade of CMVIL-10 signaling is supported by numerous studies that demonstrate evolutionarily distinct mammalian pathogens exploit the IL-10/IL-10R1 signaling pathway to subvert protective immunity. Multiple viruses (*e.g.* CMV [15], [32], Lymphocytic Choriomeningitis Virus – LCMV [33], [34], Dengue [35], [36], HIV [37], [38], human papillomavirus [39], Hepatitis B and C viruses [40], [41], [42]), bacteria (*e.g.*, *M tuberculosis* [43], *C. trachomatis* [*44*], and *L. monocytogenes* [*45*]), protozoa (*e.g.*, Leishmania [46], [47], *Plasmodium* [48]), and fungi (*e.g.*, *Paracoccidioides brasiliensis* [49]) have all coopted activation of IL-10R1 to facilitate the establishment and maintenance of a persistent infection, often in conjunction with pathogenic outcomes in the infected host [50]. While strategies may vary between organisms, they involve the immunosuppressive properties of either the host cIL-10, or a pathogen-encoded IL-10 protein, to enable microenvironments of immune privilege, tolerance, and/or immune suppression. Because of this convergent point of pathogenesis, there is now interest in prevention and therapeutic modalities that control IL-10/IL-10R1 engagement to maintain or restore immunity to various pathogens [50].

Our immunization strategy takes advantage of the protracted co-evolutionary relationship between CMV and their particular host, in which the viral IL-10 genes underwent extreme genetic drift from the cIL-10 gene. As a result, HCMV and RhCMV IL-10 proteins share only 23-26% identity with their host's cIL-10 [51]. The extent of genetic drift in the viral orthologs is highlighted by the facts that rhesus and human cIL-10 proteins share ∼95% identity, and the viral IL-10 orthologs are as divergent from each other as they are from the cIL-10 of their host. Despite sharing low amino acid identity, both CMVIL-10 and RhCMVIL-10 sequences are highly stable (95% identity) amongst different strains of HCMV and RhCMV, respectively [52]. For yet unknown reasons, virally captured cIL-10 has drifted from what was once a ‘self’ protein, expressed in the context of viral infection, to one that is now highly recognizable by the host immune system. This is highlighted by our finding that stimulation of RhCMVIL-10-Nabs does not stimulate cross-reactive antibodies against RhIL-10 that neutralize its biological activity (Fig 8).

Importantly, studies with LCMV, demonstrate that pathogen-specific immune responses are not hardwired during priming but are alterable and responsive to continuous instruction from the antigenic environment [33]. Like LCMV, MCMV does not encode a cIL-10 ortholog, but stimulates production of cIL-10 [53]. MCMV-induced up-regulation of cIL-10 impairs MHC class II antigen presentation [53], antigen-specific T cell expansion [54], and clearance of virally-infected cells from sites of persistence, such as the salivary glands (SG) [55]. Blockade of IL-10 signaling by treatment with an antibody to IL-10R1 in MCMV-infected mice leads to CD4 T cell-mediated clearance of infected cells in the SG [32], the expansion of functional MCMV-specific CD8 T cells and, notably, reduction of viral loads in the spleen and lung [56]. The observation that MCMV persistence can be reduced by altering IL-10R1 signaling is very similar to what has been shown for LCMV [33], [57]. Thus, there are precedents for determining whether CMVIL-10-based vaccine therapies in an HCMV-infected host can significantly reduce long-term parameters of HCMV infection/re-infection.

As summarized above, CMVIL-10 represents an attractive vaccine target as it exhibits potent immunosuppressive functions itself and induces cIL-10 in some cell types. In addition, because of its low sequence identity with cIL-10, it is unlikely that RhCMVIL-10-NAbs will be cross reactive with cellular IL-10. This point has now been confirmed by experiments demonstrating that our immunized animals do not exhibit antibodies that neutralize cellular RhIL-10 biological activity (Fig 8). However, a remaining problem of including CMVIL-10 in any vaccination strategy is the potential negative impact of its immunosuppressive activity, which could endanger the vaccinee and/or prevent an effective immune response to other antigens contained in the vaccine. Crystal structure analysis has provided detailed images of the HuIL-10/IL-10R1 and HuCMVIL-10/IL-10R1 interfaces required for the initial activation of the IL-10 signaling pathway [21], [27], [58]. These structural studies demonstrate cIL-10 and CMVIL-10 share common and essential molecular mechanisms to engage the IL-10R1 chain. In addition, structural studies have also characterized the neutralizing anti-IL-10 antibody (9D7) bound to cIL-10 [59]. The 9D7/cIL-10 structure illustrates that neutralization of IL-10 can occur by multiple mechanisms in addition to steric blocking of the IL-10/IL-10R1 interface [59].

Based on the principles learned from the structural analyses outline above, we have engineering non-functional RhCMVIL-10 mutants (M1, and M2) that generate antibodies that neutralize WT RhCMVIL-10 biological activity, do not cross react with cellular RhIL-10, and do not appear to harm the animals. These results provide a rationale for testing this vaccination strategy in seronegative animals, followed by challenge with RhCMV. If experiments in the RhCMV infection model are successful, the outcome of these experiments is directly translatable to human studies. In particular, residues changed in RhCMVIL-10 to create M1 and M2 are conserved in CMVIL-10. Thus, identically mutated CMVIL-10 antigens can be made for testing in phase I clinical trials.

## Materials and Methods

### Ethics statement

The University of California, Davis (UC Davis) is accredited by the Association for Assessment and Accreditation of Laboratory Animals Care (AAALAC, Animal Assurance #: A3433-01), a private, nonprofit group that promotes the humane treatment of animals in science through voluntary accreditation. UC Davis is one of more than 640 research institutions and other organizations that have earned AAALAC accreditation, demonstrating its commitment to responsible animal care and use. In addition, the CNPRC receives unannounced inspections by the U.S. Department of Agriculture, as required by the Animal Welfare Act, and inspections by the Food and Drug Administration as well. This study was carried out in strict accordance with the recommendations in the Guide for the Care and Use of Laboratory Animals of the National Institutes of Health and in accordance with the recommendations of the Weatherall report, "The use of non-human primates in research". All animal protocols were approved in advance by the Institutional Animal Care and Use Committee of UC Davis, which is fully accredited by the AAALAC. Multiple veterinarians and animal care technicians provided state-of-the-art care and research support for these studies. The animals were monitored by veterinarians and trained animal care staff every day and during all procedures. Animals were anesthetized with ketamine during blood collection and immunization to prevent any suffering. Care was taken to ensure that the animals were adequately sedated under all conditions, as assessed by the veterinarian, animal care staff, and investigators. The specific animal use protocol for this study is 15137.

### Sequence analysis and molecular modeling

Cellular and viral IL-10 amino acid sequences were aligned using clustalW2 (EMBL-EBI). All structure analyses and figures were generated using pymol [60]. Cellular IL-10 and CMVIL-10 numbering is according to the alignment of the mature cIL-10 sequence, where cIL-10 residue Ser-19 in the full length sequence is Ser-1 in this numbering scheme (Fig 2A).

### Cloning, expression, and purification of RhCMVIL-10 and RhCMVIL-10 mutants

The RhCMVIL-10 open reading frame (Fig 2A) of RhCMV strain 68-1, described by Lockridge et al. [51], was amplified by PCR and inserted into the pMTA-V5-His6 vector (Invitrogen) for expression in Drosophila S2 cells (Invitrogen), as previously described [61]. PCR was also used to construct plasmids containing N- (pMT-H6Fxa-RhCMVIL-10) and C-terminal (pMT-RhCMVIL-10-FxaH6) histidine tags for affinity purification and analysis of RhCMVIL-10 mutants. All mutations were performed using the QuikChange site-directed mutagenesis kit (Stratagene) and confirmed by DNA sequencing.

Transient and stable transfections of pMT-RhCMVIL-10 plasmids into Drosophila S2 cells were performed using the manufacturers protocols (Invitrogen). Expression was induced by addition of 0.5 mM Cu_2_SO_4_ to the media and allowed to proceed for six days. WT RhCMVIL-10 was purified by affinity chromatography using human IL-10R1 beads, as previously described by Jones et al. [21]. Large scale purification of RhCMVIL-10 mutants for vaccination studies was performed using a 2-step nickel affinity purification protocol. Expression media (1 Liter) was dialyzed against binding buffer (20 mM Tris pH 8.0, 500 mM NaCl, and 5 mM imidazole) and bound to a 5 mL column of Ni-NTA resin (Novagen). RhCMVIL-10 mutant-containing fractions were eluted from the column using bind buffer containing 200 mM imidazole. These fractions (∼30 mL) were dialyzed against binding buffer and purified again over a 0.5 mL Ni-NTA column.

### Size exclusion chromatography

Size exclusion chromatography was performed by injecting protein samples onto a 24 mL Superdex 200 gel filtration column (GE Health Care). Fractions (0.5 mL) were collected and analyzed by SDS-PAGE.

### RhCMVIL-10 Pull down assay

Drosophila media (500 µL), containing transiently expressed RhCMVIL-10 proteins, was incubated with 15 µL of IL-10R1-coupled agarose beads (affi-gel 10, BioRad) for 1 hour at 4°C. The beads were recovered from the media by spinning at 400x*g* and washed 3 times in 500 µL of wash buffer consisting of 20 mM Tris-HCL, pH 8.0, 150 mM NaCl, 1% Tween-20. Protein bound to 15 µL of the washed beads was added to sample buffer, boiled 5 minutes, and loaded onto a 12% SDS-PAGE gel. Protein was detected using coomassie staining.

### Western blotting

RhCMVIL-10 expression media (10 µL) was run on a 12% SDS-PAGE gel. RhCMVIL-10 proteins were detected by western-blotting using a primary mouse anti-tetrahis antibody (Ab) (Qiagen), diluted 1∶2000 in Tris-buffered saline with Tween-20 (TBST) and 3% bovine serum albumin (BSA), followed by a sheep anti-mouse FC horseradish peroxidase (HRP) secondary Ab, 1∶5000 dilution, in TBST and 1% non-fat milk (Amersham) for ECL detection.

### TF-1/IL-10R1 Cellular proliferation assay

TF-1/IL-10R1 cells are a human erythroleukemic cell line transfected with the human IL-10R1 chain, which causes them to proliferate upon addition of IL-10 to the media, which provides a quantitative readout of IL-10 biological activity. CIL-10 and RhCMVIL-10 mutants were dispensed into 96-well microplates (Becton Dickinson) in duplicate wells and serially diluted three-fold across the plates. 5,000 TF-1/IL-10R1 cells [31] were added to each well and incubated for 2 days at 37°C with 5% CO_2_. Viable cells were assayed using alamarBlue (Biosource International/Invitrogen). Fluorescence intensity was measured at room temperature using a POLARstar plate reader (BMG Lab technologies) at wavelengths of 544 nm excitation and 590 nm emission.

### Characterization of RhCMVIL-10 mutant activity on Rhesus PMBC

Ten-fold serially diluted RhCMVIL-10 M1 and M2 (0.1–1,000 ng) were incubated in duplicate with 4×10^5^ Ficoll-purified PBMC/well in a 96 well U-bottom plate (Falcon) for 30 minutes in a humidified 37°C incubator (5% CO_2_). LPS (from E. coli O127:B8; Sigma) was then added to the cells (5 µg/mL final concentration) followed by a 24 hour incubation at 37°C (5% CO_2_). The supernatant was collected the following day and assayed for IL-12 production by an IL-12 sandwich ELISA (described below).

### IL-12 sandwich ELISA

IL-12 secretion by LPS-activated rhesus PBMC was measured by ELISA (U-Cytech, Netherlands), according to the manufacturer's protocol with slight variations. Microtitre plates (96-well, Immulon 4 HBX, Dynex Technologies Inc.) were incubated with IL-12 p40 and p70 capture antibodies (U-Cytech) overnight at 4°C. The plates were washed 6x with PBS containing 0.05% tween (PBS-T) and then incubated with blocking buffer (PBS/1% BSA) for 1 hr at 37°C. Following the block, rhesus PBMC supernatants (100 µL/well), +/- LPS and +/-RhCMVIL-10, were incubated overnight at 4°C. The next day the plates were washed 6 times with PBS-T, followed by the addition of 100 µL/well of biotinylated anti-monkey IL-12 antibody for 1 hr at 37°C. After washing, 100 µL/well of streptavidin-HRP (U-Cytech) was added and incubated at 37°C for 1 hr. After washing, 100 µL/well TMB substrate was added per well and the plates were incubated at 25°C for 10 min. Color development was stopped by adding 50 µl/well of 0.5 M sulfuric acid. The plates were incubated at room temperature for 5 minutes, and then read at a wavelength of 450 nm on a Model 680 microplate reader (BioRad). Concentrations of IL-12 were quantified using a 2-fold serially diluted recombinant IL-12 standard that was included on each plate.

### Characterization of RhCMVIL-10/IL-10R1 interactions by SPR

All SPR experiments were performed on a Biacore T200 system (GE Healthcare) at 20 °C using HBS running buffer (10 mM Hepes (pH 7.4), 0.15 M NaCl, 0.1% P20 (GE Healthcare), and 0.1 mg/mL BSA (Sigma)). The IL-10R1-FC consisted of the extracellular region (residues 22-235, uniprot Q13651) of the human IL-10R1 chain fused to a mouse FC (IgG2a). The IL-10R1-FC was captured onto a CM5 chip surface by an anti-murine FC Ab (GE Healthcare) that was immobilized by amine coupling to the chip as described in the manufacturer's instructions. IL-10R1-FC was captured on the anti-FC surface at final densities of ∼75 RU. Kinetic interaction experiments were performed by injecting (for 90 seconds) 2-fold diluted RhCMVIL-10 over IL-10R1-FC at a flow-rate of 75 µL/min, followed by a 150 second dissociation phase. The IL-10R1-FC surface was regenerated by a 3 minute injection of 10 mM glycine, pH 1.7. The data was processed and globally fit to a bivalent analyte model (Biacore T200 Software Handbook, 2010) using Biacore T200 Software version 1.0. Estimates of RhCMVIL-10 M1 rate constants required Rmax and tc parameters to be fixed at values obtained while fitting the RhCMVIL-10 sensorgrams. For RhCMVIL-10 M2, Rmax was fixed during sensorgram fitting.

### Immunization of RhCMV-infected rhesus monkeys with RhCMVIL-10 M1 and M2

Six rhesus macaques (*Macaca mulatta*), serologically confirmed to be infected with RhCMV, were immunized with either RhCMVIL-10 M1 (N = 3) or RhCMVIL-10M2 (N = 3) using a heterologous combination of DNA and protein. DNA vectors for immunization were constructed by inserting the coding sequences for RhCMVIL-10 M1 and M2 into the Sal I and Asp 718 restriction sites of the mammalian expression vector pND, which contains the human cytomegalovirus major immediate-early promoter and enhancer, and the SV40 polyadenylation signal [62]. Recombinant plasmids (pND-RhCMVIL-10 M1 and pND-RhCMVIL-10 M2) were purified with an endotoxin-free plasmid purification kit (QIAGEN), according to the manufacturer's protocol, and the DNA concentration was determined spectrophotometrically. DNA was resuspended in PBS buffer at a concentration of 1 mg/ml and stored at −20 °C.

Animals were immunized three times with either pND-RhCMVIL-10 M1 or pND-RhCMVIL-10 M2 by both intradermal (50 µg) and intramuscular (150 µg) injection of plasmid at weeks 0, 4, and 7. Animals were boosted by two intramuscular injections of the M1 or M2 proteins at weeks 14 and 18 (50 µg) adjuvanted in Montanide ISA 720 (Seppic Inc., Fairfield, NJ), according to published protocols [63]. All animal protocols were approved in advance by the Institutional Animal Care and Use Committee of the University of California, Davis, which is fully accredited by the Association for Assessment and Accreditation of Laboratory Animal Care.

### RhcmvIL-10 ELISA

Binding antibodies against RhcmvIL-10 were characterized by ELISA by modifying a previously published protocol [64]. Briefly, 96-well microplates (Immulon 4 HBX, Dynex Technologies Inc.) were coated overnight at 4°C with nickel affinity-purified RhCMVIL-10 (12.5 ng/well) in phosphate buffered saline (PBS) (Sigma)/0.375% sodium bicarbonate (GIBCO). Each plate was subsequently washed 6 times with PBS/0.05% Tween 20 (Sigma) (PBS-T) and blocked with 300 µl/well PBS/1% bovine serum albumin (BSA) (Sigma) for 2 hours at 25°C in a temperature-controlled incubator. After washing the plates 6 times with PBS-T, 100 µl of a 1∶100 dilution of week 19 plasma (in PBS-T/1% BSA) was added to each well and incubated at 25°C for 2 hours. The plates were subsequently washed 6 times with PBS-T wash buffer and loaded with 100 µl/well of a 1∶60,000 dilution of peroxidase-conjugated goat-anti-monkey IgG (Kirkegaard & Perry Laboratories, Inc - KPL) and incubated at 25°C for 1 hour. The plates were then washed 6 times with PBS-T wash buffer and 100 µl/well of tetramethylbenzidine liquid substrate (TMB) (Sigma) was added and incubated for 30 min at 25°C. TMB color development was stopped by the addition of 50 µl/well of 0.5 M sulfuric acid. After a five minute incubation at room temperature, color development was quantified spectrophotometrically at a wavelength of 450 nm on a Model 680 microplate reader (BioRad).

### Analysis of rhesus plasma for neutralizing anti-RhCMVIL-10 antibodies

Rhesus macaque plasma samples, collected during immunization (Fig. 7), were diluted (1∶4,000) in RPMI/10% fetal bovine serum and incubated in the presence, or absence, of recombinant WT RhCMVIL-10 (0.5 ng/mL) for 3 hours at 37°C. 200 µL of the plasma +/- RhCMVIL-10 were incubated in duplicate with 4×10^5^ Ficoll-purified PBMC/well in a 96 well U-bottom plate (Falcon) for 30 minutes in a humidified 37°C incubator (5% CO_2_). LPS (from *E. coli* O127:B8; Sigma) was then added to the cells (5 µg/mL final concentration) followed by a 24 hour incubation at 37°C (5% CO_2_). Supernatants were collected the following day and the amount of IL-12 is each sample was assayed by ELISA as described above. The presence of RhCMVIL-10-NAbs was determined by comparing IL-12 levels (pg/mL) produced by LPS-activated PBMC incubated with plasma and recombinant WT RhCMVIL-10 (which at 0.5 ng/mL inhibits all IL-12 production in the absence of plasma) versus plasma only. The ratio ([ IL-12 ] ^Plasma+RhCMVIL-10^ / [ IL-12 ]^Plasma^ )*100, is plotted for each plasma sample, and represents the percent of recombinant WT RhCMVIL-10 biological activity neutralized by each plasma sample.

### Analysis for cross-neutralizing anti-RhIL-10 antibodies in vaccinated animals

Rhesus macaque week 19 plasma samples were analyzed for any cross-neutralization of RhIL-10 biological activity. The assay was performed as described for neutralization of WT RhCMVIL-10 biological activity, except that cells were incubated in 12.5 ng/ml of RhIL-10, which was the minimum concentration of RhIL-10 required to suppress IL-12 production in LPS-activated Rhesus PBMC.
